# Role of Zucchini and Its Distinctive Components in the Modulation of Degenerative Processes: Genotoxicity, Anti-Genotoxicity, Cytotoxicity and Apoptotic Effects

**DOI:** 10.3390/nu9070755

**Published:** 2017-07-14

**Authors:** Damián Martínez-Valdivieso, Rafael Font, Zahira Fernández-Bedmar, Tania Merinas-Amo, Pedro Gómez, Ángeles Alonso-Moraga, Mercedes del Río-Celestino

**Affiliations:** 1 Department of Genomics and Biotechnology, IFAPA (Andalusian Institute of Agricultural Research and Training, Fisheries, Food and Ecological Production) Center La Mojonera, Camino San Nicolás, 1 La Mojonera, 04745 Almería, Spain; damianvaldivieso@gmail.com (D.M.-V.); pedro.gomez.j@juntadeandalucia.es (P.G.); mercedes.rio.celestino@juntadeandalucia.es (M.d.R.-C.); 2 Department of Food and Health, IFAPA Center La Mojonera Camino San Nicolás, 1 La Mojonera, 04745 Almería, Spain; rafaelm.font@juntadeandalucia.es; 3 Department of Genetics, University of Córdoba, Campus Rabanales, Gregor Mendel Building, 14071 Córdoba, Spain; tania.meram@gmail.com (T.M.-A.); ge1almoa@uco.es (Á.A.-M.)

**Keywords:** cancer, carotenoid, chemoprevention, *Cucurbita*, cytotoxicity, DNA-protection, tumor cells

## Abstract

Zucchini (*Cucurbita pepo* subsp. *pepo*) is a seasonal vegetable with high nutritional and medical values. Many useful properties of this fruit are attributed to bioactive compounds. Zucchini fruits (“Yellow” and “Light Green” varieties) and four distinctive components (lutein, β-carotene, zeaxanthin and dehydroascorbic acid) were selected. Firstly, the lutein, β-carotene, zeaxanthin and dehydroascorbic acid contents were determined in these fruits. Then, in order to evaluate the safety and suitability of their use, different assays were carried out: (i) genotoxicity and anti-genotoxicity tests to determine the safety and DNA-protection against hydrogen peroxide; (ii) cytotoxicity; and (iii) DNA fragmentation and Annexin V/PI (Propidium Iodide) assays to evaluate the pro-apoptotic effect. Results showed that: (i) all the substances were non-genotoxic; (ii) all the substances were anti-genotoxic except the highest concentration of lutein; (iii) “Yellow” zucchini epicarp and mesocarp exhibited the highest cytotoxic activity (IC_50_ > 0.1 mg/mL and 0.2 mg/mL, respectively); and (iv) “Light Green” zucchini skin induced internucleosomal DNA fragmentation, β-carotene being the possible molecule responsible for its pro-apoptotic activity. To sum up, zucchini fruit could play a positive role in human health and nutrition due to this fruit and its components were safe, able to inhibit significantly the H_2_O_2_-induced damage and exhibit anti-proliferative and pro-apoptotic activities toward HL60 (human promyelocytic leukemia cells) tumor cells. The information generated from this research should be considered when selecting potential accessions for breeding program purposes.

## 1. Introduction

It is currently accepted that diet affects the overall process of carcinogenesis by different mechanisms: its constituents may contain cancer-causing substances as well as many cancer preventive agents [1]. Inappropriate dietetic habits are estimated to be the cause of more than one third of cancer deaths [2].

More than 20,000 species of plants are used in traditional medicines, alleged to be all potential reservoirs for new drugs [3] and are considered a potential source of chemical constituents with antitumor and cytotoxic activities [4]. Protective elements in a cancer prevention diet include selenium, folic acid, vitamin B-12, vitamin D, chlorophyll and antioxidants such as the carotenoids (α-carotene, β-carotene, lycopene, lutein, and cryptoxanthin) [5,6].

Summer squash (*Cucurbita pepo* subsp. *pepo*) is a seasonal vegetable that contains a number of beneficial micronutrients such as minerals, carotenoids, vitamin C, phenolic compounds, etc. [7,8,9,10]. It has been used in traditional folk medicine to treat colds and alleviate aches, due to its antioxidant/anti-radical, anti-carcinogenic, anti-inflammatory, antiviral, antimicrobial and analgesic activities [11,12,13,14,15].

Studies of genotoxicity/anti-genotoxicity and cytotoxicity are rapid methods to assess the safety and possible beneficial effects of single compounds or complex mixtures and foods [16,17,18]. The Somatic Mutation and Recombination Test (SMART) of *Drosophila melanogaster* is a suitable eukaryotic tool for genotoxicity and anti-genotoxicity studies [19] and is a one-generation test based on the loss of heterozygosity of two suitable recessive markers (*mwh* and *flr*), due to different genotoxic events (i.e., mitotic recombination, mutation and chromosomal aberration). HL60 (human promyelocytic leukemia cells) have been used in cytotoxicity assays in order to determine the tumoricide activity of some vegetable matrices [20].

There are a few studies where the cytotoxic activity of *C. pepo* has been checked. Shokrzadeh et al. [14] reported different cytotoxic activities of the *C. pepo* leaf extracts on normal and cancer cell lines. Wang et al. [15] observed a significant dose-dependent inhibitory effect against HeLa and HepG cell growth of *C. pepo* fruits extracts. Menéndez et al. [11] also reported a significant decrease of the growth of prostatic hyperplasia by extract of *C. pepo* seeds at the tested concentrations (400 and 200 mg/kg). However, no studies have been carried out to demonstrate the in vivo activity of zucchini fruit and about the mechanisms of action of their antioxidant compounds.

In the case of the genotoxic or anti-genotoxic activities of antioxidant compounds such as the ascorbic acid (the reduced form of dehydroascorbic acid), most of the reported data [21,22,23] indicate that it is anti-mutagenic. Although antioxidant protection is assumed for ascorbic acid against oxidative damage, the details of this protection are not yet completely understood. In fact, under certain conditions, ascorbic acid seems to have co-genotoxic activity instead of the normal anti-genotoxic action [24]. 

Studies about carotenoids have also been performed in relation to decrease cancer risk. Thus, the chemopreventive action of β-carotene is effective mainly at the beginning of the carcinogenic process or in the initial stages of its promotion, inhibiting the formation of pre-neoplastic lesions in some in vitro and in vivo experimental models [25,26,27]. Lutein and zeaxanthin, naturally occurring carotenoids, have shown to reduce the risk of cataracts and age-related macular degeneration [28]. Zeaxanthin is related with the reduction of melanoma cells viability [29]. Lutein caused significant DNA damage in human retinal pigment epithelial cells [30].

The aim of this work was to give an added value to the simple nutritional value of the zucchini fruit, along with some of active components, carotenoids (lutein, β-carotene, and zeaxanthin) and vitamin C, with respect to DNA integrity, and to establish the possible in vivo genotoxic and in vitro cytotoxic effects. In order to measure these properties, different assays were carried out: genotoxicity and anti-genotoxicity (in vivo analysis using the *Drosophila melanogaster* model), and cytotoxicity and pro-apoptotic activity (in vitro assays using the promyelocytic leukemia HL60 cell line). In this way, the beneficial health effects of the consumption of zucchini could be explained and used for breeding program purposes.

## 2. Materials and Methods

### 2.1. Plant Material

Two different varieties of *C. pepo* belonging to zucchini morphotype were evaluated in this work: “Light Green” (elongated in shape with light green skin) and “Yellow” (elongated in shape with yellow skin). They were representatives of zucchini commercial cultivars currently offered in the market. Seeds of these varieties were germinated on wet filter paper in Petri dishes at room temperature for 2–4 days in the dark, after which they were transplanted into rock-wool cubes (Grodan BV, Roermond, The Netherlands) in a greenhouse. Plants were transferred to 1 m large rock-wool slabs at a density of two plants/slab when developed three to four leaves. Plants were grown in a greenhouse in the Andalusian Institute of Agricultural Research and Training, Fisheries, Food and Ecological Production (IFAPA) Center in La Mojonera, Almería, Spain (36°47′19′′ N, 02°42′11′′ W, 142 m a.s.l.) from March to June 2011 following standard local cultural practices for both plant nutrition and insect pest and disease control. Six fruits of each variety were harvested at an immature stage, and processed preserving epicarp and mesocarp of each fruit separately, packaged in polypropylene plastic containers and stored at −80 °C. Sample was lyophilized using freeze drier equipment (Telstar LyoQuest, Barcelona, Catalonia, Spain) at −55 °C under vacuum (133 × 10^−3^ mbar) for 96 h per sample. Then, samples were ground and frozen at −80 °C for further extractions and biological analyses.

### 2.2. Antioxidant Compounds

The compounds used in this study were purchased from Fluka (lutein: Cat. Number 07168 and β-carotene: Cat. Number 22040, Milan, Italy), Extrasynthese (zeaxanthin: Cat. Number 0307S, Genay, France) and Sigma-Aldrich (ascorbic acid: Cat. Number 255564 and dehydroascorbic acid: Cat. Number 261556, St. Louis, MO, USA). The carotenoids were dissolved in ethanol prior to addition to the corresponding culture media, i.e., in water for fly treatment, or in RPMI (Roswell Park Memorial Institute) 1640 medium for HL60 cell culture at the time of the experiment. The final concentration of ethanol was 1% in the culture media.

### 2.3. Determination of the Carotenoid Content

All manipulations were performed in ice and under subdued artificial light conditions with headspaces of containers flushed with oxygen free nitrogen to help prevent carotenoid degradation. Individual carotenoid concentration was determined by reverse phase high performance liquid chromatography (HPLC) after saponification following the methods described by Martínez-Valdivieso et al. [10]. The carotenoids were extracted from the rehydrated sample with 5 mL ethanol containing 1 mg/mL butylated hydroxytoluene (BHT) using a Polytron homogenizer (Polytron Kinematica, Newark, NJ, USA). Samples were saponified in order to hydrolyze esterified carotenoids that mightcomplicate the chromatographic determinations [31]. One milliliter of a 40% KOH methanolic solution (*w*/*v*) was added to each tube, and the samples were saponified for 10 min at 85 °C. The samples were cooled in an ice bath, and 2 mL of ice-cold water was added. The suspensions were extracted twice with 2 mL of hexane by vigorous vortexing followed by a 2000 rpm centrifugation for 10 min at room temperature. The upper hexane layers were pooled and evaporated to dryness in a Savant SpeedVac apparatus and resuspended (Waltham, MA, USA). Immediately before injection the carotenoids were dissolved in 800 µL of an acetonitrile/methanol/dichloromethane (45:20:35 *v*/*v*/*v*) solution, filtered through a 0.22 µm polytetrafluoroethylene (PTFE) syringe filter (Millipore, Billerica, MA, USA) directly to sample vials, and 10 µL were injected into the chromatograph. The initial mobile phase consisted of acetonitrile/methanol (97:3, *v*/*v*/*v*) containing 0.05% (*v*/*v*) triethylamine. We used a linear gradient of dichloromethane from 0 to 10% in 20 min at the expense of acetonitrile, and then the dichloromethane was kept constant at 10% until the completion of the runs. The flow rate was 1.0 mL/min while the column temperature was 30 °C. The analyses were carried out on a HPLC apparatus equipped with binary pump, in-line vacuum degasser, auto sampler injector, a Waters Symmetry C18 column (4.6 mm × 150 mm, 5 µm particle size), (Waters, Milford, MA, USA) and a 996 diode array detector (Waters, Milford, MA, USA) supported by the Empower chromatography manager computing system (Waters) was used to detect colored carotenoids at 450 nm. Compounds were identified by comparison of retention times, co-injection with known standards, and comparison of their ultraviolet (UV)-visible spectra with authentic standards. Quantification was carried out by external standardization. Full standard curves were constructed with five different concentrations for each carotenoid in triplicate. The curves passed through or were very near the origin, were linear and bracketed the concentrations expected in the samples. Results were expressed on a dry weight (DW) basis.

Once the content of the selected antioxidant compounds was evaluated in the epicarp and the mesocarp of *C. pepo* fruit, the genotoxicity, cytotoxicity and apoptosis assays were performed.

### 2.4. Extraction and Analysis of Vitamin C

The vitamin C analysis was carried out with freeze dried lyophilized samples stored at −80 °C. Five grams of samples were homogenized in 10 mL of MeOH/H_2_O (5:95) plus citric acid (21 g/L) with EDTA (0.5 g/L) and 4 mM NaF. Homogenates were then filtered through cheesecloth and C18 Sep-Pak cartridges (Waters, Milford, MA, USA). Ascorbic acid (AA) and dehydroascorbic acid (DHA) contents were determined following the methods described by Zapata and Dufour [32,33]. HPLC analyses were performed after derivatization of DHA into the fluorophore 3-(1,2-dihydroxyethyl) furol [3,4-b]quinoxaline-1-one (DFQ), with 1,2-phenylenediamine dihydrochloride (OPDA). Samples of 20 μL were analyzed by using a Merck-Hitachi (Tokyo, Japan). The analyses were carried out on a HPLC apparatus equipped with binary pump, in-line vacuum degasser, autosampler injector, a Waters and a 996 diode array detector (Waters, Milford, MA, USA) supported by the Empower chromatography manager computing system (Waters). Separations of DFQ and AA were achieved on a Kromasil 100 C18 column (250 mm × 4 mm; 5 μm particle size; Tecnokroma, Barcelona, Spain). The mobile phase was MeOH/H_2_O (5:95, v/v) containing 5 mM cetrimide and 50 mM potassium dihydrogen phosphate at pH 4.5. The flow rate was 0.9 mL/min. The detector wavelength was initially set at 348 nm and after elution of DFQ, the wavelength was manually shifted to 261 nm for AA detection. Standard solutions, column conditioning and derivatization procedures have been previously described by Gil et al. [34].

### 2.5. Genotoxicity and Anti-Genotoxicity Tests

The principles and basic procedures for the *Drosophila* wing spot test have been described by Graf et al. [19], and in previous works of our group [16,17]. Two strains of flies carrying wing genetic markers on the left arm of chromosome 3: *multiple wing hair* (*mwh*, 3-0.3) and *flare* (*flr^3^*, 3-38) are used. The transheterozygous larvae were obtained by crossing *mwh*/*mwh* males and *flr*^3^/TM3 (*Third Multiple 3*), *Bd^S^*(*Beaded serrate*) virgin females. Hybrid eggs derived from crossing optimally fertile flies were collected over an 8 h period. Larvae emerged 72 ± 4 h later were cleaned from remaining feeding medium with distilled water, and subsequently transferred to treatment vials. These vials contained 0.85 g of *Drosophila* Instant Medium (Formula 4–24, Carolina Biological Supply, Burlington, NC, USA) wetted with 4 mL of the epicarp and mesocarp of *C. pepo* and their antioxidant compounds solutions at physiological concentrations for *Drosophila melanogaster*: 0.25 and 8 mg/mL of epicarp and mesocarp of each variety and the correspondent concentrations of pure compounds based on the previously detailed determination (0.039 and 0.615 μM for lutein, 0.0003 and 0.0689 μM for β-carotene, 0.0001 and 0.105 μM for zeaxanthin, and 0.003 and 0.107 mM for dehydroascorbic acid). Concurrent negative controls with the solvent alone (water) and positive controls with hydrogen peroxide (120 mM) were also run.

**Figure 1:**
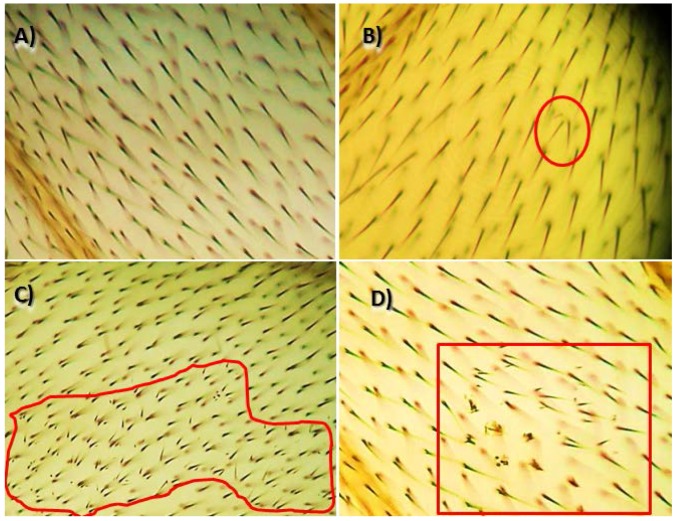
Types of spots found in the *Drosophila melanogaster* spot wing test: (**A**) wild phenotype (normal trichomes); (**B**) simple *mwh* spot; (**C**) large *mwh* spot; and (**D**) twin spot.

Anti-genotoxicity tests were carried out by mixing the mutagen (hydrogen peroxide, 120 mM) with the compounds solution. After emergence, adult flies were collected from the treatment vials and stored in 70% ethanol. The wings of the flies were removed under a stereomicroscope using a pair of entomological tweezers, similar number of males and females-wings were mounted in Faure’s solution on microscope slides and inspected, under 400× magnification, for the presence of clones of cells. The mutant clones were classified into three types: (1) small single spots, containing one or two cells; (2) large single spots, containing three or more cells; and (3) twin spots, containing adjacent *mwh* and *flr*^3^ cells [19]. The appearance of twin spots indicated the recombinogenic activity of the chemotherapeutic agent (Figure 1).

### 2.6. Data Evaluation and Statistical Analysis

Differences between quality compounds present in epicarp and mesocarp from “Yellow” and “Light Green” zucchini were assessed by Student´s *t*-test. Data normality was tested prior to analysis. SPSS Version 10.0 software was used to perform all statistical analyses (SPSS, Chicago, IL, USA).

The frequencies of spots per fly of treated series were compared with its corresponding negative control series (water) to analyze Somatic Mutation and Recombination Test (SMART) data. For maximum power, statistical analyses were done exclusively for the total number of spots recovered. The non-parametric *U* test of Mann, Whitney and Wilcoxon was used to avoid weak positive and inconclusive results on SMART and to achieve a well-defined statistical diagnosis whether a given treatment should be regarded as genotoxic or not [35]. The inhibition percentages (IP) were calculated using the total spots per wing with the following formula [16,36]:

IP = ((Genotoxine alone − Genotoxine plus active compound)/Genotoxine alone) × 100

### 2.7. Cell Culture and Cytotoxicity Assay

HL60 cells were cultured in suspension at 37 °C in RPMI 1640 medium (BE12-167F, BioWhittaker, Verviers, Belgium) supplemented with 10% fetal bovine serum (DE14-801F, BioWhittaker), Glutamine (G7513, Sigma) and an antibiotic-antimycotic solution (A5955, Sigma) in a humidified atmosphere containing 5% CO_2_. Cultured HL60 cell (2 × 10^5^ cells/mL) was treated for 72 h with epicarp, mesocarp and different compounds at the concentrations in which they are found in the fruit: epicarp (0.015–0.25 mg/mL) and mesocarp (0.015–0.5 mg/mL) from “Yellow” *C. pepo*, and epicarp and mesocarp (0.031–0.5 mg/mL) from “Light Green” *C. pepo*, lutein (0.0039–0.25 μM), β-carotene (0.023–0.466 μM), zeaxanthin (0.022–0.879 μM), dehydroascorbic acid (0.0341–0.681 mM). A mix of β-carotene, zeaxanthin and dehydroascorbic acid at the same concentrations above indicated was also prepared to check if the cytotoxic effects were in additive or synergic way.

The reactivity of trypan blue (93595, Sigma), a vital dye, is based on the fact that the chromophore is negatively charged and does not interact with the cell unless the membrane is damaged. Therefore, all the cells that exclude the dye are viable. Non-viable cells stained purple-violet, whereas viable ones remained unstained. Trypan blue was added to cell cultures at a 1:1 ratio, and 20 μL of cell suspension was loaded into a Neubauer chamber. The cells were counted under an inverted microscope at 100× magnification. After the incubation period, a growth curve was established and IC_50_ values (concentration of test compound causing 50% inhibition of cell growth) were estimated. Curves are expressed as survival percentage with respect to controls growing at 72 h. All data represent the average of at least three independent experiments.

### 2.8. Assessment of Pro-Apoptosis by DNA Fragmentation and Annexin V-PI

The HL60 tumoral cell line was used with the aim of analyse the apoptotic induction. HL60 cells (1.5 × 10^6^ cells) were treated with the same concentrations of the different compounds for 5 h. A non-treated cells control was included for each experiment. For DNA fragmentation, treated cells were collected, were centrifuged at 4000 rpm for 5 min and washed with PBS. The DNA was extracted using a commercial kit (MBL 243, Dominion mbl, Córdoba, Spain) and the resulting total DNA was treated with RNAse in order to eliminate false positive for 30 min at 37 °C. Samples of 1.5 μg of DNA were mixed with loading buffer and loaded onto a pre-solidified 2% agarose gel containing ethidium bromide. The agarose gels were run at 50 V for 90 min in TBE (Tris/Borate/EDTA) buffer and then observed and photographed under UV light. For Annexin V-PI test, Annexin V Apoptosis Detection Kit (Canvax Biotech S.L., Cordoba, Spain) was used. Briefly, cells were resuspended in 100 μL of Binding buffer solution 1× and subsequently, 5 μL AnnexinV-FITC (Fluorescein isothiocyanate) and 10 μL propidium iodide were added, and samples were incubated for 10 min at room temperature in darkness, and flow cytometry (Beckman Coulter Inc., Brea, CA, USA) was then used to analyze the rate of apoptosis.

## 3. Results

### 3.1. Quantitation of Antioxidant Compounds

The mean content values of the different active compounds in epicarp and mesocarp of the two summer squash varieties are listed in Table 1. The two varieties differed in the content of all the compounds analyzed. The “Yellow” zucchini carotenoid content was significantly higher than “Light Green” zucchini in both tissues, except zeaxanthin in mesocarp. It should be noted that the lutein epicarp content of “Yellow” zucchini is seven times higher than “Light Green” zucchini. Further, in the latter variety, β-carotene was not detected in epicarp. Dehydroascorbic acid content of “Light Green” zucchini was similar in both tissues, but mesocarp of “Yellow” zucchini was tenfold higher than epicarp. In addition, dehydroascorbic acid mesocarp content of “Yellow” zucchini was significantly higher than “Light Green” zucchini.

### 3.2. Genotoxicity and Anti-Genotoxicity Analysis of C. pepo and Their Components

The results of chronic treatment of *D. melanogaster* transheterozygous larvae with zucchini epicarp and mesocarp and their different antioxidant compounds, along or combined with one fixed concentration (120 mM) of hydrogen peroxide in the *Drosophila* wing spot assay (SMART) are presented in Table 2 and Table 3. The positive control hydrogen peroxide (H_2_O_2_) behaved as a genotoxin inducing oxidative stress and DNA damage, giving the expected result. It exhibited a total mutation rate (0.52 mutant clones/wing), which quadrupled the negative control (distilled water) rate (0.13). This ratio ensures the accuracy of the genotoxicity and anti-genotoxicity concurrent assays [37].

No compound resulted genotoxic in the SMART at the tested concentrations (Table 2). There was no significant increase in the numbers of small single spots and total spots in the case of zucchini epicarp and mesocarp, and single compounds at the studied doses. Mutation rates were even lower than the negative water control. The only case with high but non-significant mutation rate was the highest concentration of lutein with 0.45 total spots/wing for 0.615 μM. Zeaxanthin and dehydroascorbic acid induced a non-significant increase of mutation frequencies at the highest tested concentrations (0.15 total spots/wing at 0.105 μM, and 0.18 at 0.107 mM, respectively) after larval feeding. Thus, in the wing spot test in vivo model, carotenoids and dehydroascorbic acid are not genotoxic.

Table 3 shows the results of the anti-genotoxicity assays. The results obtained in the combined treatment of larvae with zucchini epicarp and mesocarp (0.25 and 8 mg/mL) showed non-significant differences when compared to the negative control. Then, the SMART test showed that zucchini epicarp and mesocarp, and bioactive compounds were able to detoxify the genotoxic activity of hydrogen peroxide although no dose effect was observed in general (Table 3). The mutation frequency in β-carotene, zeaxanthin and dehydroascorbic acid was even smaller than that obtained with the negative control. Only the highest concentration of lutein showed significant differences with the negative control (0.47 for 0.615 μM).

**Figure 2:**
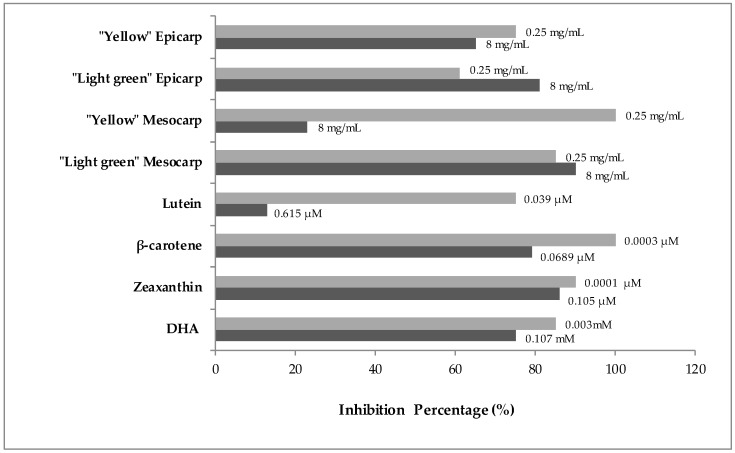
Inhibition percentages (IP) of zucchini epicarp and mesocarp and its bioactive compounds against H_2_O_2_-induced damage in the *Drosophila* wing spot test.

The inhibition percentage (IP) of genotoxicity by epicarp and mesocarp of “Yellow” zucchini was 75% and 100% at 0.25 mg/mL and 65% and 23% at 8 mg/mL, respectively. In the case of the epicarp and mesocarp of “Light Green” zucchini a dose-dependent IP was observed being the highest detoxifying activity by 90 % at 8 mg/mL of epicarp. The fruit antioxidant compounds would act as anti-mutagens against hydrogen peroxide in SMART. The lowest concentration of “Yellow” zucchini mesocarp (0.25 mg/mL) showed the highest IP (100 %). The bioactive zucchini selected compounds exhibited an IP that ranged from 13% to 100% (on average 75%) (Figure 2).

It should be noted that the SMART test is suitable to detect the anti/pro-mutagenic effects of some chemicals [2,16,17,24,39]. The results reported here are of interest when investigating the different ways in which summer squash and their antioxidant compounds can interfere in vivo with the mechanisms of genotoxic agents.

### 3.3. Effects on Tumoral Growth of HL60 Cells and Apoptosis

Cultured mammalian cells provide an important tool for evaluating the cytotoxicity of compounds with potential therapeutic activity [40]. Trypan blue assessment was used to check the cytotoxicity of epicarp and mesocarp of two summer squash varieties and the main antioxidant compounds of the fruit.

**Figure 3:**
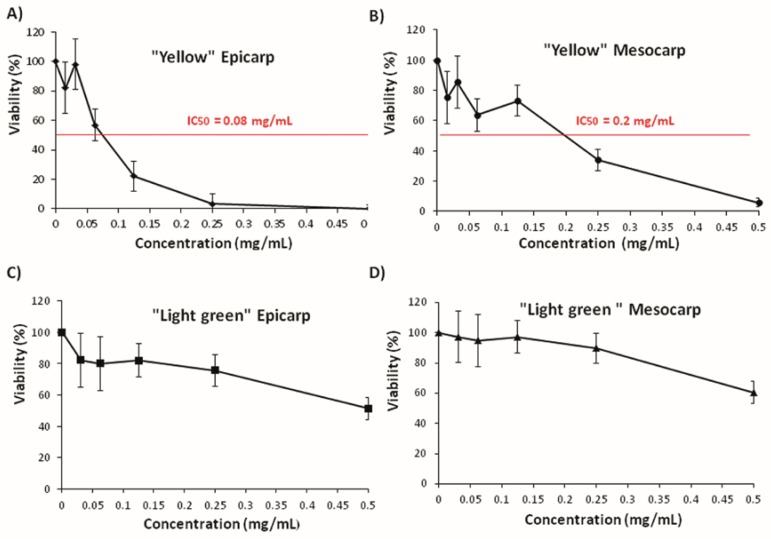
Effects of “yellow” and “Light green” zucchini epicarp ((**A**) and (**C**) respectively) and mesocarp ((**B**) and (**D**) respectively) on viability of HL60 (human promyelocytic leukemia cells) tumoral cells. Cell viability was assessed after 72 h by trypan blue exclusion test assay. The data are expressed as percentage with respect to control (mean ± SD from three independent experiments). The IC_50_ values (Inhibitory concentration 50) are showed in red. SD: standard deviation.

**Figure 4:**
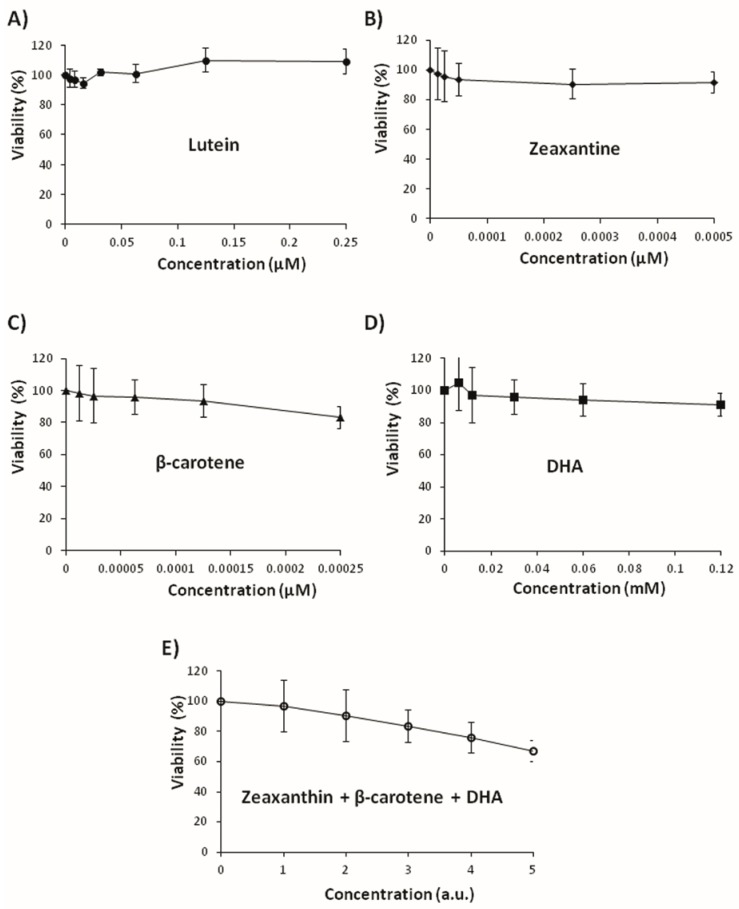
Effects of lutein (**A**), zeaxanthin (**B**), β-carotene (**C**), dehydroascorbic acid (DHA) (**D**) and a mix of compounds (zeaxanthin, β-carotene and DHA) (**E**) on viability of HL60 cells. Cell viability was assessed after 72 h by trypan blue exclusion test assay. The data are expressed as percentage with respect to control (mean ± SD from three independent experiments). a.u.: arbitrary units.

A wide range of concentrations was used for every compound. Figure 3 and Figure 4 show the relative tumor growth inhibition activity of the assayed substances. The results are expressed as survival percentage with respect to the controls (% tumoral viability). The shapes of the curves were different for each case. Epicarp and mesocarp of “Yellow” zucchini showed the most negative slope and the highest tumoricidal effect in the trypan blue exclusion assay (IC_50_ = 0.08 mg/mL and 0.2 mg/mL, respectively). Epicarp and mesocarp of “Light Green” zucchini also showed a negative slope, but not reaching IC_50_. The rest of compounds assayed did not reach IC_50_ at the tested concentrations, although slight cytotoxic activities were observed in some of them (zeaxanthin, dehydroascorbic acid, β-carotene and the mix of the compounds) (Figure 4). We did not observe any cytotoxic effect of lutein at the chosen concentrations. Taking these observations together, it appears that the cytotoxic activity of zucchini epicarp and mesocarp cannot be related to the antioxidant effect of a single antioxidant compound, but rather to the total interactions between different compounds of such a complex mixture.

**Figure 5:**
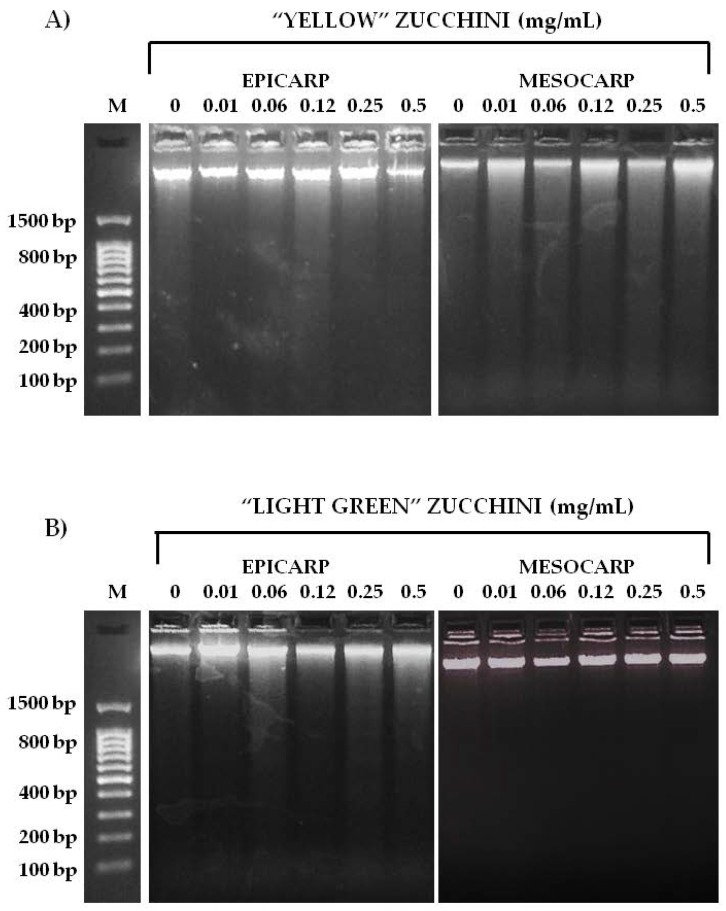
Nucleosomal DNA fragmentation assay: (**A**) “Yellow” zucchini; and (**B**) “Light Green zucchini. HL-60 cells were exposed to various concentrations of zucchini epicarp and mesocarp for 5 h. DNA was extracted from cells and was subject to 2% agarose gel electrophoresis at 50 V for 90 min. M: DNA size marker.

**Figure 6:**
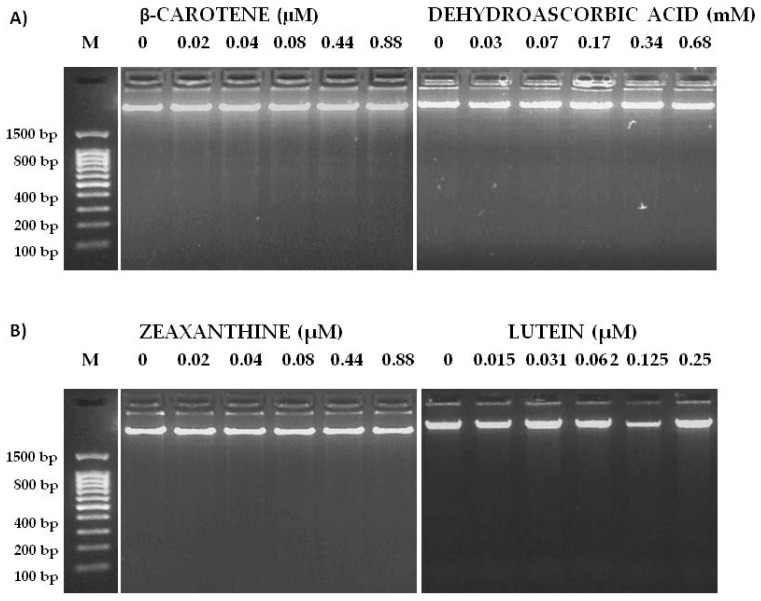
Nucleosomal DNA fragmentation assay. HL-60 cells were exposed to various concentrations of β-carotene, dehydroascorbic acid (**A**), zeaxanthin and lutein (**B**) for 5 h. DNA was extracted from cells and was subject to 2% agarose gel electrophoresis at 50 V for 90 min.

**Figure 7:**
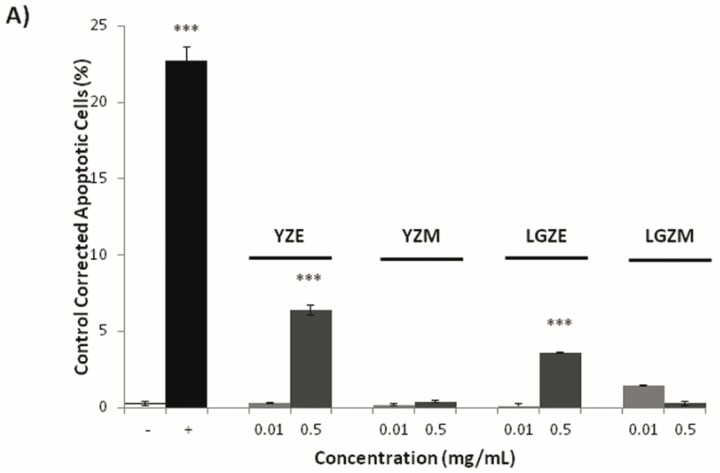

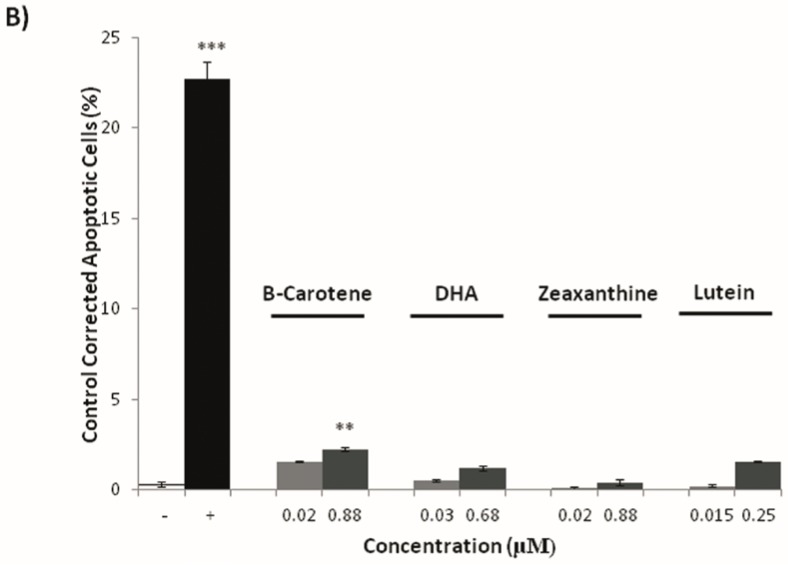
Analysis of cell death apoptotic way in HL60 cells by Annexin V-PI staining after 5 h of treatment with different concentrations of: YZE (yellow zucchini epicarp), YZM (yellow zucchini mesocarp), LGZE (light green zucchini epicarp), and LGZM (light green zucchini mesocarp) (**A**); and different carotenoids and DHA (dehydroascorbic acid) (**B**). -: negative control; +: positive control; ** *p* ≤ 0.01; *** *p* ≤ 0.001.

Once cytotoxicity assays of zucchini epicarp and mesocarp and some of its antioxidant compounds were performed, a visible assay of DNA fragmentation was carried out in order to investigate whether the mechanism undergoing the cytotoxicity was mediated via apoptosis. The degradation of genomic DNA into oligonucleosomal fragments is a hallmark of apoptosis. This DNA fragmentation endpoint was analyzed by conventional agarose gel electrophoresis. The HL60 cell line was treated for 5 h with de same concentrations as in the trypan blue cytotoxicity assay. As shown in Figure 5 and Figure 6, the highest concentrations of “Light Green” zucchini epicarp showed a slight internucleosomal fragmentation ladder pattern of DNA for HL60 treated cells. This did not occur in the control and some lower concentrations. A dose-dependent effect is suggested. “Yellow” zucchini, “Light green” zucchini mesocarp, β-carotene, lutein, zeaxanthin and DHA did not show any internucleosomal fragmentation ladder pattern. The results of Annexin V/Propidium iodide (PI) assay (Figure 7) showed significant differences between the negative control and the highest concentrations of Yellow zucchini Epicarp, Light Green zucchini epicarp and β-carotene samples, indicating an induced apoptosis.

## 4. Discussion

Common prevention strategies include avoiding exposure to known cancer-causing agents, enhancement of host-defense mechanisms against cancer, lifestyle modifications and chemoprevention [41]. Chemoprevention is essential for the reversion, inhibition and prevention of cancer, and, optimally, requires the use of non-toxic agents that inhibit molecular steps during the carcinogenic pathway [42]. Prevention by dietary phytochemicals is an important approach in cancer management [43].

The first step of the present work was to characterize the different active compounds in zucchini epicarp and mesocarp matrices. As the results show, both varieties differ greatly in bioactive compound contents in both tissues. The antioxidant contents exhibited by the samples were included in the variation interval found in the literature for *C. pepo*. Lutein and β-carotene contents in epicarp and mesocarp fall within the range reported in previous studies [10], but our data show lutein mesocarp content in “Yellow” zucchini (362.7 mg/kg dry weight) is higher than reported in mesocarp by Tadmor et al. [44] (143.2 mg/kg dry weight, assuming 92% of moisture). El-Qudah [45] analyzed the lutein, β-carotene and zeaxanthin contents in zucchini fruit and their results were lower than ours for the three carotenoids (23.4, 1.46 and 0.41 mg/kg dry weight, respectively). In relation to vitamin C, in our study the loss of ascorbic acid was offset by the simultaneous increase of dehydroascorbic acid (a biologically active vitamin). As reported in previous studies, ascorbic acid (vitamin C) is a very unstable compound and it easily transforms into its oxidized form, dehydroascorbic acid [46]. Ascorbic acid can be easily degraded, depending on many variables such as pH, temperature, light, and presence of enzymes, oxygen, and metallic catalyzers [47]. Another important factors influencing vitamin C retention in fresh-cut vegetables are the cutting mechanism and drying methods used. Cutting the product may promote the loss of vitamin C as a result of the increase of the surface area in contact with oxidizing agents such as oxygen that favor the action of ascorbate peroxidase (APX), the main enzyme responsible for degradation of vitamin C [48]. In relation to drying methods, although freeze-drying is considered one of the main techniques to preserve phytochemicals, a negative effect on ascorbic acid, and other compounds such as total phenolics and carotenoids have been previously reported in different foodstuff [49,50,51].

The SMART test has been shown to be a good predictor of safe/harmful substances due to the ability of *Drosophila* larvae to activate many procarcinogens and the possibility for individually treating thousands of somatic cells [52]. The main objective of this assay was to evaluate the safety of zucchini epicarp and mesocarp for the first time, as well as their major active components (lutein, β-carotene, zeaxanthin and dehydroascorbic acid) with respect to the lack of induction of genetic damage. The wing spot test for zucchini tissues and their components resulted non-significant at the assayed concentrations when compared to the water control.

Anti-genotoxic agents generally exhibit desirable chemotherapeutic effects that could be efficient in the strategy of cancer control [53]. Hydrogen peroxide was used as a positive control in SMART because it is reported that it generates DNA damage through oxygen-radical mechanisms, gene mutation, chromosomal aberration and DNA single-strand breaks [54]. Nevertheless, it is not known whether the interactions that can occur between oxidative elements (H_2_O_2_) and other bioactive compounds (lutein, β-carotene, zeaxanthin and DHA) act mutually. Previous studies have reported the relation between bioactive compounds such as carotenoid, ascorbic acid and phenolic contents and their antioxidant activity evaluated by DPPH (1,1-diphenil-2-picrylhydrazyl) in fruit extracts of *Cucurbita* spp. [55,56]. The concentrations of the selected compounds were shown to be anti-genotoxic in the SMART test of *Drosophila melanogaster*. The strongest inhibition ability was detected with the lowest concentration of “Yellow” zucchini mesocarp (0.25 mg/mL) and the lowest concentration of β-carotene (0.0003 μM) against the genotoxic effects of H_2_O_2_ in the imaginal discs of *Drosophila* (100% for both).

The cytotoxicity assessment is an in vitro bioassay needed in the evaluation of the chemopreventive effects of a substance as a fast, non-expensive and informative first step of screening. The human cell line HL60 provides a reliable model to study the cytotoxic effect of chemopreventive substances and the mechanisms underlying this potential activity [39].

Some examples of similar results in relation to the cytotoxicity of *C. pepo* in the in vitro cytogenetic assay with different cell lines have been reported. Wang et al. [15] observed a significant dose-dependent inhibitory effect against HeLa and HepG cell growth using ethanolic extracts of *C. pepo* fruits. Menéndez et al. [11] remarked a significant decrease of the prostatic growth using a lipophilic extract of *C. pepo* seeds at doses of 400 and 200 mg/kg. Shokrzadeh et al. [14] used hydro-alcoholic extracts of C. *pepo* leaves on normal (Chinese hamster ovarian cells (CHO) and rat fibroblast) and cancer (liver hepatocellular cells HepG2 and colon carcinoma cell CT26) cell lines with the following rank of inhibition ability: CHO < fibroblast < CT26 < HepG2 (being the lowest and the highest IC_50_ for HepG2: 132.6 μg/mL; and for fibroblast: 293.2 μg/mL) cell lines. Other studies revealed that aerial parts of other *Cucurbita* species such as *C. maxima* also possess significant anticancer activity in the Ehrlich ascites carcinoma model in mice which may be due to its cytotoxicity and antioxidant properties [4].

Anti-proliferative properties and induction of apoptosis in tumor cells are suitable when the health-protecting effect of antioxidants is assessed [53]. Apoptosis plays critical roles in the development and maintenance of homeostasis, in multicellular organisms. During apoptosis, intracellular contents are not released and potentially harmful inflammatory responses are prevented. Apoptotic cell death is characterized by a DNA fragmentation into 180–200 pb nucleosomal units [17] and at the molecular level the apoptotic way can also be monitored trough the Annexin V/PI assay [57]. The DNA fragmentation assay was only able to detect apoptosis in HL60 exposed to highest concentrations of “Light Green” zucchini epicarp. Nevertheless, the Annexin assay provides evidence that the cells exposed to some concentrations of compounds suffer cell death via apoptosis and therefore their activity could be catalogued like an anti-proliferative action. Precisely, β-carotene and epicarp of both types of zucchini significantly induced apoptosis. We hypothesize that β-carotene could be the molecule responsible in part for the chemopreventive effects of yellow zucchini epicarp. The compounds that promote apoptosis should become an important addition to the arsenal of target-specific drugs in the fight against cancer. The results of our study have shown that treatment of HL60 cells with epicarp of “Light Green” zucchini, indicating the involvement of apoptosis.

In the present study, we demonstrated that a complex mixture such as zucchini epicarp and mesocarp, inhibited proliferation in the human promyelocytic HL60 cell line. In addition, our results provide a basic knowledge about phytochemicals interactions with biological systems that may be useful for the design of functional foods. Sharoni et al. [58] hypothesized that a single micronutrient cannot replace the power of the concerted action of multiple agents derived from a diet rich in fruits and vegetables.

Carotenoids have been studied widely to demonstrate if these colorful compounds can decrease the cancer risk [6]. Dias et al. [59] obtained similar results in the SMART test for β-carotene using higher concentrations than ours (1, 2 and 4 mg/mL): β-carotene was not genotoxic and was able to detoxify up to 95 % of the mutation induced by doxorubicin. Zhang and Omaye [60] demonstrated that the antioxidant and pro-oxidant effects of β-carotene are dependent of both, the level of oxygen and the β-carotene concentration. For most biological tissues, when the oxygen level is low, β-carotene, as other carotenoids, becomes important as an antioxidant [61]. The chemopreventive action of β-carotene is effective mainly in the beginning of the carcinogenic process or in the initial stages of its promotion, inhibiting the formation of pre-neoplastic lesions in experimental models in vitro and in vivo [25,26,27]. The ability of β-carotene to inhibit cell growth has been established in several tumor cells including melanoma [62], prostate [63], colon [64], lung, breast and oral mucosa cancer [65]. In our study, β-carotene did not show cytotoxicity (0.5 μM) which agrees with Sacha et al. [66] who used β-carotene in the concentrations available in vivo (10 μM) and did not affect leukemic cell lines (HL60). Higher doses (ID_50_ = 27 μM) of β-carotene used in HL60 cells by Palozza et al. [67] modulated cell cycle progression and induced apoptosis in a dose-dependent manner; nevertheless, the delay in cell growth by β-carotene was highly coincident with the increased intracellular ROS production and oxidized glutathione content induced by the β-carotene. On the other hand, Sacha et al. [66] also informed that β-carotene stimulated apoptosis in HL60 by modulating the expression of the regulatory genes at 10 μM concentration. This pigment at 10 μM and 50 μM also stimulated apoptosis in B16F cells (melanoma) [68] and in the MCF-7 cell line [69], respectively. Although epidemiologic studies have demonstrated that a high intake of vegetables containing β-carotene decreases the risk of cancer, different studies have revealed that β-carotene supplementation to smokers resulted in a high incidence of lung cancer, it could alternatively behave as an antioxidant or as a pro-oxidant molecule, depending on its redox potential and on the cellular environment [67]. Studies tend to agree that the overall intake of carotenoids is more protective than a high intake of a single carotenoid [6] as β-carotene may be a marker for the intake of fruits and vegetables, but it does not have a powerful protective effect in isolated pharmacological doses. Thus, a variety of fruits and vegetables is still a better anti-cancer strategy than just using a single vegetable with high content in a specific carotenoid.

With respect to the rest of the carotenoids (lutein and zeaxanthine), the cytotoxic effects of zeaxanthin on two human uveal melanoma cell lines (SP6.5 and C918) were studied and compared to effects on normal ocular cells (uveal melanocytes, retinal pigment epithelial cells, and scleral fibroblasts): zeaxanthin reduced the cell viability of melanoma cells in a dose-dependent manner (10, 30 and 100 μM), with IC_50_ at 40.8 and 28.7 μM in SP6.5 and C918 cell lines, respectively. Zeaxanthin did not affect the viability of normal ocular cells even at the highest levels tested (300 μM), suggesting that zeaxanthin has a selectively cytotoxic effect on melanoma cells; in addition, zeaxanthin (30 μM) induced apoptosis in melanoma cells [29].

Previous work on lutein indicated that it is able to induce significant DNA damage in a dose- and time-dependent manner in human retinal pigment epithelial cells (ARPE-19) at a concentration ranging from 10 to 50 μM [30]. Lutein has an anti-proliferative activity on HeLa, HCT116 and Hep2 cancer cell lines [70,71,72]. On the other hand, similar negative effects to ours were obtained by Fernández-Bedmar and Alonso-Moraga [16] when HL60 cells were treated with this carotenoid. Roberts et al. [73] demonstrated that when the human lens epithelial cells (HLE B-3) were pretreated with lutein (20 μM added 2 h prior to irradiation with ultraviolet A (UVA)), the singlet oxygen quencher-lutein significantly protected against fullerol photodamage, phototoxic damage decreased by half.

Other studies have been conducted with rats where the toxicity of zeaxanthin and lutein present in different commercial preparations has been evaluated, and no toxicologically relevant findings were noted in any study. Moreover, in these studies, the results of zeaxanthin mutagenicity testing in the *Salmonella typhimurium* model did not reveal any genotoxicity [28,74,75], and neither in *Escherichia coli* [75]. In the same way, lutein was not genotoxic in the *Drosophila melanogaster* genetic model [16].

Low blood levels of ascorbic acid are detrimental to health and vitamin C is correlated with overall good health and cancer prevention [6]. Dehydroascorbic acid did not show cytotoxicity in our studies at 0.7 mM, but Park et al. [76] informed that L-ascorbic acid (the reduced form of dehydroascorbic acid) showed cytotoxicity and induced apoptosis in in vitro cultures of malignant cells (HL60, NB4 and NB4-R1), when using higher concentrations than ours (0.25–1 mM). Moreover, dehydroascorbic acid (0.5–1 mM) was tested for its protective effect against phototoxic damage induced by fullerol, resulting in some activity against metabolic damage [73].

The results obtained in genotoxicity and anti-genotoxicity assays of zucchini epicarp and mesocarp and its different bioactive compounds (lutein, β-carotene, zeaxanthin and dehydroascorbic acid) indicated that zucchini is not genotoxic and safe. “Light Green” and “Yellow” zucchini showed anti-mutagenic capacity, but the capacity of the “Light Green” variety was higher on average. This difference in the anti-genotoxic activities could be due to the different content of bioactive molecules. With the current data, authors suggest that lutein could be the responsible molecule for the different anti-mutagenic activity of two varieties, as although lutein is not genotoxic, it is at the limit of significance. Moreover lutein was anti-genotoxic at the lowest concentration, but at highest concentration enhances the genotoxicity of hydrogen peroxide. With respect to the cytotoxicity results, “Light Green” zucchini showed in the present study lower activity than “Yellow” zucchini. The differences in the carotenoid’s content could partially explain the different cytotoxic activities, as can be observed in Figure 3, where the inhibition of the tumoral growth by the mix of the three compounds is equivalent to the addition of the inhibitions of the separate individual compounds (zeaxanthin, β-carotene and dehydroascorbic acid).

Further works will be continued in our studies to find other possible candidate compounds responding to the cytotoxicity of zucchini. Thus, a previous study demonstrated the presence of secondary metabolites (flavanoids, quinones, coumarins, carbohydrates, glycosides and phytosteroids) with potential antioxidant and cytotoxic activities in fruit extracts of bitter squash (*Cucurbita digitata*) [77]. Other compounds with cytotoxic potential are the cucurbitacins, which are a class of cucurbitane-type tetracyclic triterpenoids mainly produced by plants of the family of *Cucurbitaceae*. They are renowned for their bitter taste, but also possess a broad range of potent biological activities, driving largely from their cytotoxic and anti-tumor properties [78]. Although the cytotoxicity of cucurbitacins was known before 1800 *Anno Domini* (AD), very little is known about the mechanism of the effect of cucurbitacins at the cellular and molecular level, which accounts for the relatively slow advance in cucurbitacin-based anti-cancer drug discovery. It has also been reported that cucurbitacin E exhibits specific cytotoxicity against prostate carcinoma explants, and the mechanism underlying this process is to induce disruption of the action and vimentin cytoskeleton in prostate carcinoma cells [79].

## 5. Conclusions

*C. pepo* is an important crop and source of human food around the world. Our results confirmed the safety, anti-genotoxicity and chemopreventive potential of zucchini and some of its compounds using the SMART test in vivo model and the cytotoxicity HL60 cells in vitro model. Anti-genotoxicity assays indicated that all the concentrations showed protective anti-genotoxic activity with different inhibition percentages (ranging from 11% to 100% inhibition) in combined treatments with hydrogen peroxide as a genotoxicant, except for the highest concentration of lutein. The leukemic cell line HL60 provides an important model for studying the mechanisms and relationships between cytotoxicity, apoptosis and antitumor efficacy of different substances and compounds. Epicarp and mesocarp of “Yellow” zucchini exhibited the highest cytotoxic activity (IC_50_ > 0.08 mg/mL and 0.2 mg/mL, respectively); the rest of the bioactive compounds assayed did not reach IC_50_ at the tested concentrations.

The result of the present investigation is quite encouraging and it explores the potential anticancer activity of zucchini, being probably due to its direct cytotoxic effect which is further enhanced by its antioxidant properties. The zucchini fruit showed promising results, and could play a beneficial role in human nutrition and general health. We conclude that *C. pepo* and their components were safe, able to inhibit significantly the H_2_O_2_-induced damage and exhibit anti-proliferative and pro-apoptotic properties toward HL60 tumor cells. The information generated from in vitro and in vivo studies about the zucchini phytochemical profile that contributes to improve the consumer’s health is essential for selecting potential accessions for breeding program purposes.

## Figures and Tables

**Table 1:** Epicarp and mesocarp mean content of lutein, β-carotene, zeaxanthin and dehydroascorbic acid from two zucchini varieties (“Yellow” and “Light green”) expressed in mg/kg dry weight.

	Yellow	Light
Epicarp		
Lutein	1036.9 ^a^	135 ^b^
β-carotene	99.5 ^a^	n.d. ^b^
Zeaxanthin	18.6 ^a^	1.7 ^b^
DHA	369.3 ^b^	592 ^a^
Mesocarp		
Lutein	362.7 ^a^	63.2 ^b^
β-carotene	31.6 ^a^	4.0 ^b^
Zeaxanthin	1.9 ^a^	1.7 ^a^
DHA	3200 ^a^	683.3 ^b^

Means within the same row followed by the same letter are not significantly different using Student’s test at *p* < 0.05. n.d.: not detected.

**Table 2:** Genotoxicity (single treatment) of epicarp and mesocarp of two varieties of *C. pepo* and its active compounds (lutein, β-carotene, zeaxanthin and dehydroascorbic acid) in the *Drosophila* Wing Spot Test.

Clones Per Wing (Number of Spots) ^1^						
Compound	*N*	Small Spots (1–2 Cells) *m* = 2	Large Spots (>2 Cells) *m* = 5	Twin Spots *m* = 5	Total Spots *m* = 2	Mann-Whitney Test ^3^
**Negative Control (H_2_O)**	40	0.08 (3) ^2^	0.00 (0)	0.05 (2)	0.13 (5)	
**Epicarp**						
**Yellow (mg/mL)**						
0.25	40	0.15 (6)	0.00 (0)	0.03 (1)	0.18 (7)	n.s.
8	40	0.00 (0)	0.00 (0)	0.03 (1)	0.03 (1)	n.s.
**Light Green (mg/mL)**						
0.25	40	0.10 (4)	0.03 (1)	0.00 (0)	0.13 (5)	n.s.
8	40	0.05 (2)	0.00 (0)	0.00 (0)	0.05 (2)	n.s.
**Mesocarp**						
**Yellow (mg/mL)**						
0.25	40	0.08 (3)	0.03 (1)	0.00 (0)	0.10 (4)	n.s.
8	40	0.05 (2)	0.00 (0)	0.00 (0)	0.05 (2)	n.s.
**Light Green (mg/mL)**						
0.25	40	0.03 (1)	0.03 (1)	0.00 (0)	0.05 (2)	n.s.
8	40	0.03 (1)	0.03 (1)	0.00 (0)	0.05 (2)	n.s.
**Single Compounds**						
**Lutein (μM)**						
0.039	40	0.15 (6)	0.08 (3)	0.00 (0)	0.23 (9)	n.s.
0.615	40	0.33 (13)	0.08 (3)	0.05 (2)	0.45 (18)	n.s.
**β-carotene (μM)**						
0.0003	36	0.08 (3)	0.00 (0)	0.00 (0)	0.08 (3)	n.s.
0.0689	40	0.03 (1)	0.03 (1)	0.00 (0)	0.05 (2)	n.s.
**Zeaxanthin (μM)**						
0.0001	40	0.05 (2)	0.00 (0)	0.00 (0)	0.05 (2)	n.s.
0.105	40	0.10 (4)	0.03 (1)	0.03 (1)	0.15 (6)	n.s.
**DHA (mM)**						
0.003	40	0.10 (4)	0.03 (1)	0.00 (0)	0.13 (5)	n.s.
0.107	40	0.15 (6)	0.03 (1)	0.00 (0)	0.18 (7)	n.s.

^1^ Statistical diagnosis by Frei and Würgler [38]. *N*: number of wings; ^2^ Number of clones or spots per wing; ^3^ The inconclusive results were resolved using the non-parametric U-Test Mann-Whitney and Wilcoxon. n.s.: non-significant. DHA: Dehydroascorbic acid. *m*: multiplication factor.

**Table 3:** Anti-genotoxicity (combined treatment) of epicarp and mesocarp of two varieties of *C. pepo* and its active compounds (lutein, β-carotene, zeaxanthin and dehydroascorbic acid) in the *Drosophila* Wing Spot Test.

Clones Per Wing (Number of Spots) ^1^						
Compounds	*N*	Small Spots (1–2 Cells) *m* = 2	Large Spots (>2 Cells) *m* = 5	Twin Spots *m* = 5	Total Spots *m* = 2	Mann-Whitney Test ^3^
**Negative Control (H_2_O)**	40	0.08 (3) ^2^	0.00 (0)	0.05 (2)	0.13 (5)	
**Positive Control (H_2_O_2_ 120 mM)**	40	0.38 (15)	0.13 (5)	0.03 (1)	0.52 (21)	*
**Epicarp**						
**Yellow (mg/mL)**						
0.25	16	0.13 (5)	0.00 (0)	0.00 (0)	0.13 (5)	n.s.
8	40	0.15 (6)	0.03 (1)	0.00 (0)	0.18 (7)	n.s.
**Light Green (mg/mL)**						
0.25	40	0.13 (5)	0.05 (2)	0.03 (1)	0.20 (8)	n.s.
8	40	0.05 (2)	0.03 (1)	0.03 (1)	0.10 (4)	n.s.
**Mesocarp**						
**Yellow (mg/mL)**						
0.25	18	0.00 (0)	0.00 (0)	0.00 (0)	0.00 (0)	n.s.
8	40	0.38 (15)	0.00 (0)	0.03 (1)	0.40 (16)	n.s.
**Light Green (mg/mL)**						
0.25	40	0.05 (2)	0.03 (1)	0.00 (0)	0.08 (3)	n.s.
8	40	0.05 (2)	0.00 (0)	0.00 (0)	0.05 (2)	n.s.
**Single Compounds**						
**Lutein (μM)**						
0.039	32	0.06 (2)	0.06 (2)	0.00 (0)	0.13 (4)	n.s.
0.615	32	0.25 (8)	0.16 (5)	0.06 (2)	0.47 (15)	*
**β-carotene (μM)**						
0.0003	12	0.00 (0)	0.00 (0)	0.00 (0)	0.00 (0)	n.s.
0.0689	38	0.08 (3)	0.03 (1)	0.00 (0)	0.11 (4)	n.s.
**Zeaxanthin (μM)**						
0.0001	40	0.03 (1)	0.00 (0)	0.03 (1)	0.05 (2)	n.s.
0.105	30	0.07 (2)	0.00 (0)	0.00 (0)	0.07 (2)	n.s.
**DHA (mM)**						
0.003	40	0.08 (3)	0.00 (0)	0.00 (0)	0.08 (3)	n.s.
0.107	40	0.10 (4)	0.03 (1)	0.00 (0)	0.13 (5)	n.s.

^1^ Statistical diagnosis by Frei and Würgler [36]. *N*: number of wings; ^2^ Number of clones or spots per wing; ^3^ The inconclusive and positive results were resolved using the non-parametric U-Test Mann-Whitney and Wilcoxon. n.s.: non-significant; *: significant (*p* ≤ 0.05). *m*: multiplication factor.
